# Phenotype-Based High-Throughput Classification of Long QT Syndrome Subtypes Using Human Induced Pluripotent Stem Cells

**DOI:** 10.1016/j.stemcr.2019.06.007

**Published:** 2019-08-01

**Authors:** Daisuke Yoshinaga, Shiro Baba, Takeru Makiyama, Hirofumi Shibata, Takuya Hirata, Kentaro Akagi, Koichi Matsuda, Hirohiko Kohjitani, Yimin Wuriyanghai, Katsutsugu Umeda, Yuta Yamamoto, Bruce R. Conklin, Minoru Horie, Junko Takita, Toshio Heike

**Affiliations:** 1 Department of Pediatrics, Graduate School of Medicine, Kyoto University, 54 Kawahara-cho, Shogoin, Sakyo-ku, Kyoto 606-8507, Japan; 2 Department of Cardiovascular Disease, Graduate School of Medicine, Kyoto University, Kyoto, Japan; 3 Gladstone Institute of Cardiovascular Disease, University of California San Francisco, San Francisco, USA; 4 Departments of Medicine and Pharmacology, University of California San Francisco, San Francisco, USA; 5 Department of Cardiovascular and Respiratory Medicine, Shiga University of Medical Science, Otsu, Japan

**Keywords:** long QT syndrome, induced pluripotent stem cell, phenotype-based diagnosis, multi-electrode array, genome editing

## Abstract

For long QT syndrome (LQTS), recent progress in genome-sequencing technologies enabled the identification of rare genomic variants with diagnostic, prognostic, and therapeutic implications. However, pathogenic stratification of the identified variants remains challenging, especially in variants of uncertain significance. This study aimed to propose a phenotypic cell-based diagnostic assay for identifying LQTS to recognize pathogenic variants in a high-throughput manner suitable for screening. We investigated the response of LQT2-induced pluripotent stem cell (iPSC)-derived cardiomyocytes (iPSC-CMs) following I_Kr_ blockade using a multi-electrode array, finding that the response to I_Kr_ blockade was significantly smaller than in Control-iPSC-CMs. Furthermore, we found that LQT1-iPSC-CMs and LQT3-iPSC-CMs could be distinguished from Control-iPSC-CMs by I_Ks_ blockade and I_Na_ blockade, respectively. This strategy might be helpful in compensating for the shortcomings of genetic testing of LQTS patients.

## Highlights

• I_Ks_ blockade showed a smaller response of delayed repolarization in LQT1-iPSC-CMs
• I_Kr_ blockade showed a smaller response of delayed repolarization in LQT2-iPSC-CMs
• I_Na_ blockade markedly ameliorated prolonged repolarization in LQT3-iPSC-CMs
• Ion-channel blockade response distinguished LQTS subtypes with high accuracy

## Teaser

The methods presented by the authors allowed recognition of long QT syndrome (LQTS) subtypes 1, 2, and 3 using specific ion-channel current blockade with a combination of patient-derived iPSCs and a multi-electrode array system. This strategy might potentially compensate for the shortcomings of genetic testing for LQTS, especially in patients who have variants of unknown significance or no identified mutations.

## Introduction

Long QT syndrome (LQTS) is caused by hereditary cardiac channelopathies characterized by a prolonged QT interval and abnormal T-wave morphology on electrocardiograms and capable of precipitating malignant arrhythmia (i.e., Torsade de Pointes [TdP]), resulting in syncope and sudden death (Moss, 2003). Genetic tests are currently utilized to assist treatment selection and prognostication (Napolitano et al., 2005); however, the advent of high-output sequencing techniques using next-generation sequencing has allowed identification of an extremely large number of variants from both patients and healthy individuals. Given the extreme clinical importance of identifying pathogenic variants among those of uncertain significance (i.e., VUSs) (Horie, 2016), a phenotype-based high-throughput diagnostic test is required to identify clinically relevant genetic abnormalities.

Giudicessi and Ackerman (2013) suggested “current-centric” classification of LQTS-susceptibility genes, which is reasonable in terms of phenotype-based diagnosis and subsequent treatment selection. However, current-centric classification is difficult in clinical settings. Although the use of provocative tests, including exercise-stress tests and drug-infusion tests, has been proposed to predict the LQTS genotypes, it remains difficult to appropriately diagnose and manage decisions based on their results (Priori et al., 2013).

Human induced pluripotent stem cell (iPSC) technology is promising for cell transplantation and disease modeling for diagnosis, investigation of disease mechanisms, and identification of new drugs (Hamazaki et al., 2017, Inoue and Yamanaka, 2011, Takahashi et al., 2007). Because iPSCs retain the genetic information of the cells from which they are derived, cells differentiated from iPSCs can potentially recapitulate the phenotypic variation of each donor. Therefore, iPSC-derived cardiomyocytes (iPSC-CMs) potentially play a key role in various fields of regenerative medicine (Egashira et al., 2011) and might represent a powerful diagnostic tool for LQTS.

Here, we demonstrated that iPSC-CMs can be applied to phenotypic, cell-based, high-throughput screening for recognition and classification of LQTS by using iPSCs from patients with LQTS types 1, 2, and 3, which account for ∼90% of all LQTS.

## Results

### Clinical Phenotype of LQTS Patients Enrolled in this Study and Genetic Mutations

**Figure 1:**
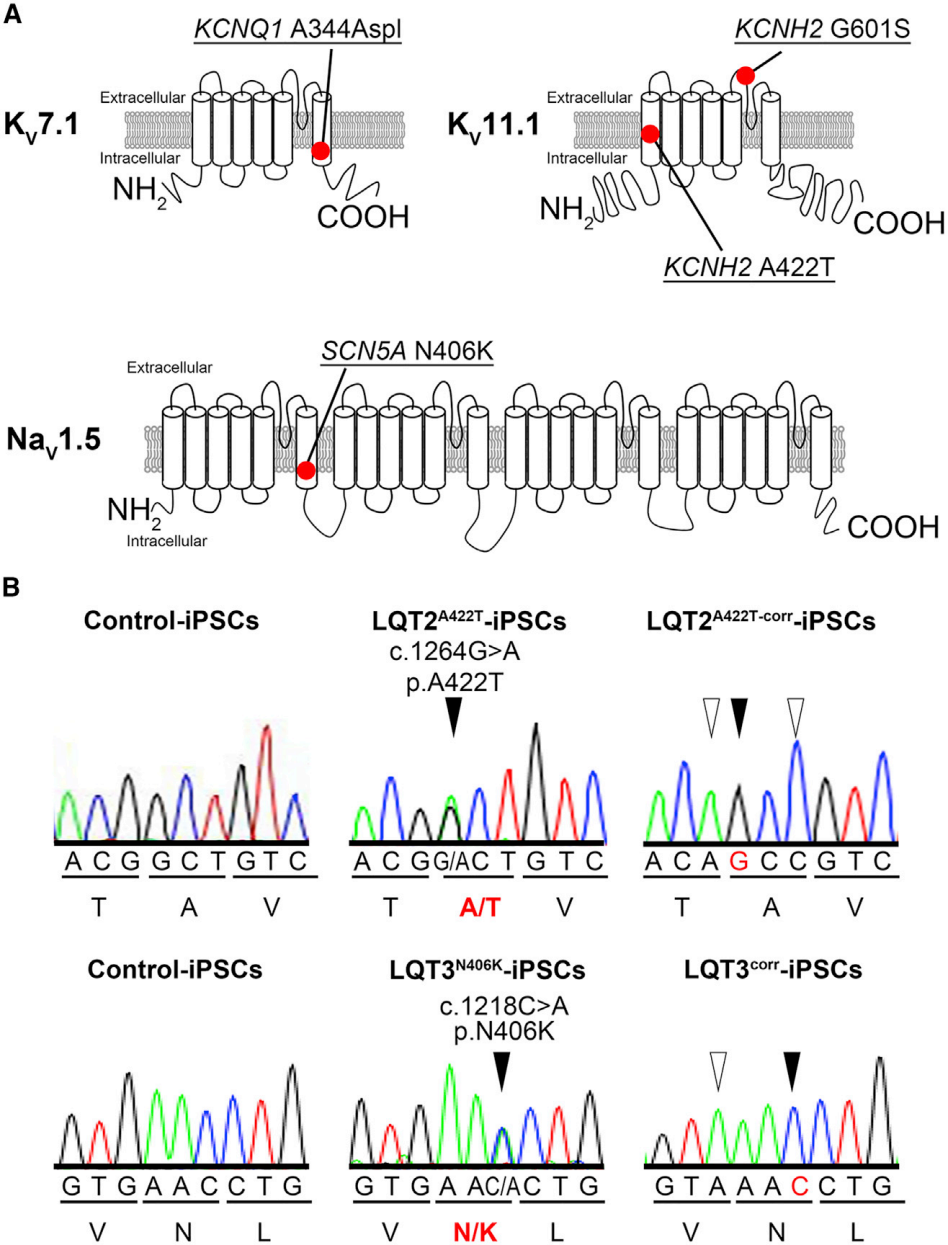
Introduction of iPSC Lines Used in This Study (A) Summary of topologies of mutations of iPSC lines used in this study. LQT1^A344Aspl^ carries a heterozygous *KCNQ1* mutation (c.1032C > A, p.A344Aspl); LQT2^A422T^ and LQT2^A422T-corr^ are the isogenic pair harboring the heterozygous *KCNH2* mutation (c.1264G > A, p.A422T) and the corrected sequence, respectively; LQT2^G601S^ carries a heterozygous *KCNH2* mutation (c.1801G > A, p.G601S); and LQT3^N406K^ and LQT3^corr^ are the isogenic pair harboring the heterozygous *SCN5A* mutation (c.1218C > A, p.N406K) and the corrected sequence, respectively. (B) Sequence analysis of PCR-amplified genomic DNA of the isogenic pair of LQT2^A422T^ and LQT2 ^A422T-corr^ and of LQT3^N406K^ and LQT3^corr^, respectively. The gene-corrected cell lines harbor several silent mutations (white arrow head) to avoid further digestion by CRISPR/Cas9.

All patients enrolled in this study were symptomatic, except for an LQT2 patient harboring potassium voltage-gated channel subfamily H member 2 (*KCNH2*) p.G601S (Table 1). All mutations were located in the transmembrane or pore domains, thereby causing heterozygous missense mutations in *KCNH2* and sodium voltage-gated channel alpha subunit 5 (*SCN5A*) and splicing errors in potassium voltage-gated channel subfamily Q member 1 (*KCNQ1*) (Figure 1A). The sequence of gene-corrected LQT2^A422T^-iPSCs (LQT2^A422T-corr^-iPSCs) and LQT3^N406K^-iPSCs (LQT3^corr^-iPSCs) was confirmed by Sanger sequencing (Figure 1B).

**Table 1:** Information on Patients Enrolled in the Present Study

LQTS Type	Mutation	Age (years)	Sex	Corrected QT Interval	Symptom
LQT1	*KCNQ1*	11	Male	424 ms	Cardiac arrest
	A344Aspl			500 ms on exercise	Ventricular fibrillation
LQT2	*KCNH2*	53	Female	493 ms	Syncope
	A422T				
	*KCNH2*	14	Female	480 ms	Asymptomatic
	G601S				
LQT3	*SCN5A*	20	Female	522 ms	Sudden death
	N406K				
Healthy Control		46	Male	443 ms	None
		36	Female	No QT prolongation	None

### LQT2^A422T^- and LQT2^G601S^-iPSC-CMs Display Smaller Responses to I_Kr_ Blockade

**Figure 2:**
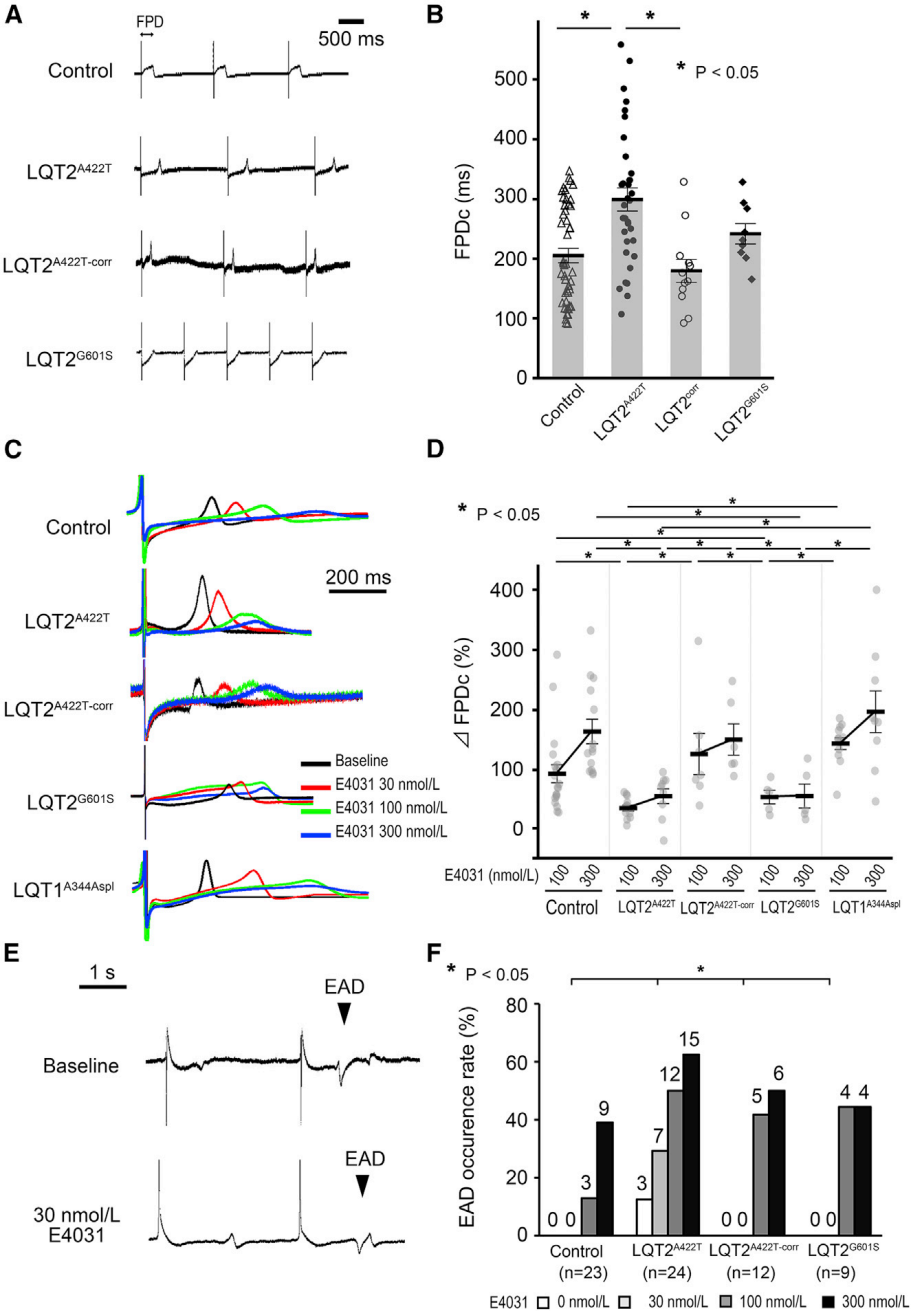
Functional Analysis of iPSC-CMs Using MEA Following I_Kr_ Blockade (A) Representative traces of FP in Control-, LQT2^A422T^-, LQT2^A422T-corr^-, and LQT2^G601S^-iPSC-CMs. (B) FPDc baseline data in Control-, LQT2^A422T^-, LQT2^corr^-, and LQT2^G601S^-iPSC-CMs (independent experiments, n = 45, 35, 12, and 9 from independent differentiation experiments, n = 16, 11, 6, and 3, respectively; mean ± SEM; p = 0.001; one-way ANOVA). ^∗^p < 0.05, Fisher's LSD post hoc test. (C) Representative traces of the FPD following administration of 30 nmol/L (red), 100 nmol/L (green), and 300 nmol/L (blue) E4031. (D) Averaged FPDc ratio before and after E4031 treatment (%ΔFPDc) in Control-, LQT2^A422T^-, LQT2^A422T-corr^-, LQT2^G601S^-, and LQT1^A344Aspl^-iPSC-CMs. FPDc prolongation upon treatment with 100 and 300 nmol/L E4031 was smaller in LQT2^A422T^- and LQT2^G601S^-iPSC-CMs than in Control-, LQT2^corr^-, or LQT1^A344Aspl^-iPSC-CMs (independent experiments, n = 23, 24, 12, 9, and 12 from independent differentiation experiments, n = 8, 8, 6, 3, and 3 in Control-, LQT2^A422T^-, LQT2^A422T-corr^-, LQT2^G601S^-, and LQT1^A344Aspl^-iPSC-CMs, respectively; mean ± SEM; p = 0.025; two-way repeated measures ANOVA). ^∗^p < 0.05, Fisher's LSD post hoc test. (E) Examples of EADs. EAD documented in a sample of LQT2^A422T^-iPSC-CMs before E4031 treatment (upper) and following 30 nmol/L E4031 treatment (lower). (F) Percentage of samples in which EADs occurred in Control-, LQT2^A422T^-, LQT2^A422T-corr^-, and LQT2^G601S^-iPSC-CMs. The number on top of each bar shows the number of arrhythmic events. ∗p < 0.05; Pearson's Chi-square test. See also Figure S1.

We hypothesized that I_Kr_ contribution to repolarization could be indirectly evaluated by assessing the field-potential (FP) response to I_Kr_ blockade. At baseline, the corrected LQT2^A422T^-iPSC-CM FP duration (FPDc) was significantly longer than that in Control- and LQT2 ^A422T-corr^-iPSC-CMs (301.0 ± 15.7 ms, 202.2 ± 10.8 ms, and 180.2 ± 20.9 ms, respectively; p < 0.05) (Figures 2A and 2B). To verify our hypothesis, we blocked I_Kr_ using E4031 in LQT2^G601S^- and LQT1^A344Aspl^-iPSC-CMs. Figure 2C displays representative FP traces in each CM group treated with 30, 100, and 300 nmol/L E4031. To evaluate the contribution of blocked I_Kr_ to CM repolarization, we calculated %ΔFPDc of iPSC-CMs except for those with early afterdepolarizations (EADs). Upon administration of 100 and 300 nmol/L E4031, the %ΔFPDc of LQT2^A422T^- and LQT2^G601S^-iPSC-CMs was significantly smaller than that of Control-, LQT2A422T-corr-, and LQT1A344Aspl-iPSC-CMs (Figure 2D). These results suggested that %ΔFPDc upon I_Kr_ blockade reflected the density of I_Kr_. Regarding the FPDc value, LQT1^A344Aspl^-iPSC-CMs showed the most prolonged FPDc among the five iPSC-CM lines (Figure S1A).

During the prolonged FPD by I_Kr_ blockade, EADs were recorded in each cell line (Figure 2E), revealing that, upon less than 100 nmol/L E4031, the occurrence rate of EADs was significantly higher in LQT2^A422T^-iPSC-CMs than in Control-, LQT2^A4222T-corr^-, LQT2^G601S^-, or LQT1^A344Aspl^-iPSC-CMs (Figure 2F). These data suggested that LQT2^A422T^-iPSC-CMs were more vulnerable to arrhythmic events.

### Patch-Clamp Results Show Reduced I_Kr_ and Attenuated Response to I_Kr_ Blockade in LQT2^A422T^-iPSC-CMs

**Figure 3:**
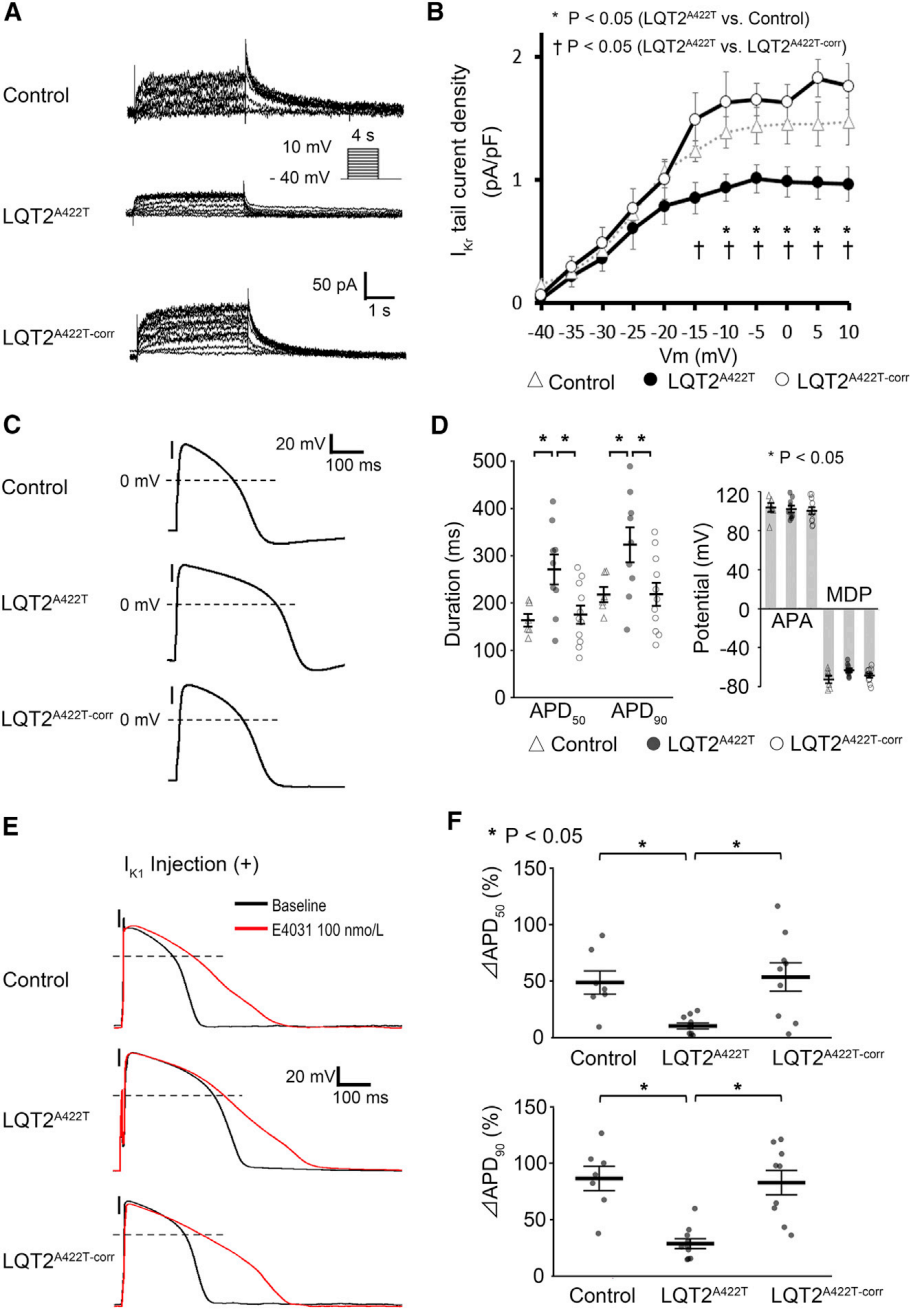
Electrophysiological Properties of iPSC-CMs and AP Response to I_Kr_ Blockade (A) Representative current traces of the I_Kr_ in Control-, LQT2^A422T^-, and LQT2^A422T-corr^-iPSC-CMs. (B) Average current-voltage relationships for peak tail currents in Control-, LQT2^A422T^-, and LQT2^A422T-corr^-iPSC-CMs (independent experiments, n = 8, 6, and 5 from independent differentiation experiments, n = 4, 4, and 3, respectively; mean ± SEM; p = 0.029; two-way repeated measures ANOVA). ^∗^p < 0.05, Fisher's LSD post hoc test for Control versus LQT2^A422T^; †p < 0.05, LQT2^A422T-corr^ versus LQT2^A422T^. (C) Representative traces of AP with 1-Hz pacing. (D) APD_50_ and APD_90_ in Control-, LQT2^A422T^-, and LQT2^A422T-corr^-iPSC-CMs (independent experiments, n = 6, 9, and 11, from independent differentiation experiments, n = 5, 6, and 5, respectively; mean ± SEM; p = 0.010 and p = 0.025 for APD_50_ and APD_90_, respectively; one-way ANOVA). ^∗^p < 0.05, Fisher's LSD post hoc test for APD_50_ and APD_90_. (E) Representative AP traces changed by I_Kr_ blockade. (F) Percentage of APD prolongation after E4031 treatment (%ΔAPD) at 1-Hz pacing in each cell line. %ΔAPD_50_ and %ΔAPD_90_ in Control-iPSC-CMs and LQT2^A422T-corr^-iPSC-CMs versus LQT2^A422T^-iPSC-CMs (independent experiments, n = 7, 10, and 10 from independent differentiation experiments, n = 5, 5, and 6 in Control-, LQT2^A422T^-, and LQT2^A422T-corr^-iPSC-CMs, respectively; mean ± SEM; p = 0.001; two-way repeated measures ANOVA). ^∗^p < 0.05, Fisher's LSD post hoc test. See also Figure S2. APA, action potential amplitude; MDP, maximum diastolic potential.

To investigate the I_Kr_ in differentiated CMs, we performed patch-clamp electrophysiological analysis, with I_Kr_ current detected as an E4031-sensitive current (Figure 3A). I_Kr_ tail current densities in LQT2^A422T-corr^-iPSC-CMs increased significantly as compared with those in LQT2^A422T^-iPSC-CMs according to the whole-cell patch-clamp method (peak I_Kr_ density: LQT2^A422T^, 1.01 ± 0.11 pA/pF; LQT2 ^A422T-corr^, 1.85 ± 0.16 pA/pF) (Figure 3B), indicating that gene correction normalized the reduced I_Kr_ observed in LQT2^A422T^-iPSC-CMs. We then evaluated action potential duration (APD) to assess the contribution of I_Kr_ to repolarization (Figure 3C), finding that the APD_90_ in Control-, LQT2^A422T^-, and LQT2 ^A422T-corr^-iPSC-CMs was 215.8 ± 16.2 ms, 321.1 ± 37.0 ms, and 216.4 ± 24.4 ms, respectively (p < 0.05), whereas the AP amplitude and maximum diastolic potential (MDP) did not differ significantly (Figure 3D).

To confirm I_Kr_ blockade-mediated FPD prolongation, we evaluated APD prolongation upon I_Kr_ blockade. Because MDP elevation due to I_Kr_ blockage disturbed correct APD measurement in some samples (Figure S2A), MDP was fixed at approximately –80 mV by introducing an artificial I_K1_ current using the dynamic clamp method (Table 2; Figure S2B), thereby allowing the evaluation of APD prolongation in the presence of I_Kr_ blockade (Figures 3E and S2A). APD_90_ prolongation induced by treatment with a higher concentration of E4031 was significantly shorter in LQT2^A422T^-iPSC-CMs than in Control- and LQT2 ^A422T-corr^-iPSC-CMs (28.4 ± 4.4% versus 85.8 ± 10.7% and 82.1 ± 10.7%, respectively; p < 0.05), with the APD_50_ showing a similar pattern of prolongation (10.4 ± 2.6% versus 48.6 ± 10.2% and 53.4 ± 12.5%, respectively; p < 0.05). These responses to E4031 were compatible with those observed in FPD experiments (Figure 3F). These data rigorously supported the multi-electrode array (MEA) results, showing that the response to I_Kr_ blockade reflected I_Kr_ density.

**Table 2:** Parameters of Action Potentials under I_K1_ Injection with Dynamic Clamp in Each Cell Line

	Baseline				100 nmol/L E4031 Administration			
	Control	LQT2^A422T^	LQT2^corr^	p Value	Control	LQT2^A422T^	LQT2^corr^	p Value
No. of cells	8	14	13		7	10	10	
APD_50_ (ms)	192.8 ± 12.4^a^	261.3 ± 21.6	197.7 ± 17.4^a^	0.026	278.7 ± 22.8^b^	277.9 ± 29.5	301.9 ± 51.7	0.883
APD_90_ (ms)	236.0 ± 14.0^a^	310.2 ± 22.1	237.8 ± 19.4^a^	0.019	423.7 ± 25.1^b^	384.8 ± 37.5	412.4 ± 58.4^b^	0.833
MDP (mV)	−81.4 ± 1.0	−82.2 ± 0.4	−81.1 ± 0.7	0.408	−78.5 ± 1.6	−81.6 ± 0.6	−80.3 ± 0.7	0.102
APA (mV)	119.4 ± 2.1	124.3 ± 1.7	119.0 ± 1.8	0.066	121.8 ± 3.3	121.2 ± 2.4	120.0 ± 2.2	0.886
Cm (pF)	65.9 ± 7.9	66.5 ± 9.3	64.2 ± 10.3	0.990	61.6 ± 7.6	66.5 ± 11.5	64.2 ± 13.4	0.838
Injected I_K1_ (pA/pF)	1.0 ± 0.1	1.1 ± 0.1	1.0 ± 0.1	0.874	1.0 ± 0.1	1.0 ± 0.1	1.0 ± 0.1	0.955

p Values were calculated using one-way ANOVA.

APA, action potential amplitude; Cm, membrane capacitance; MDP, maximum diastolic potential; pA, picoampere; pF, picofarad.

a p < 0.05; Fisher's LSD post hoc test for Control or LQT2^corr^ versus LQT2^A422T^.

b p < 0.05; parameters of the same cell line before and after administration of 100 nmol/L E4031 were statistically compared using an unpaired Student's t test.

### LQT1^A344Aspl^-iPSC-CMs Display an Attenuated Response to I_Ks_ Blockade

**Figure 4:**
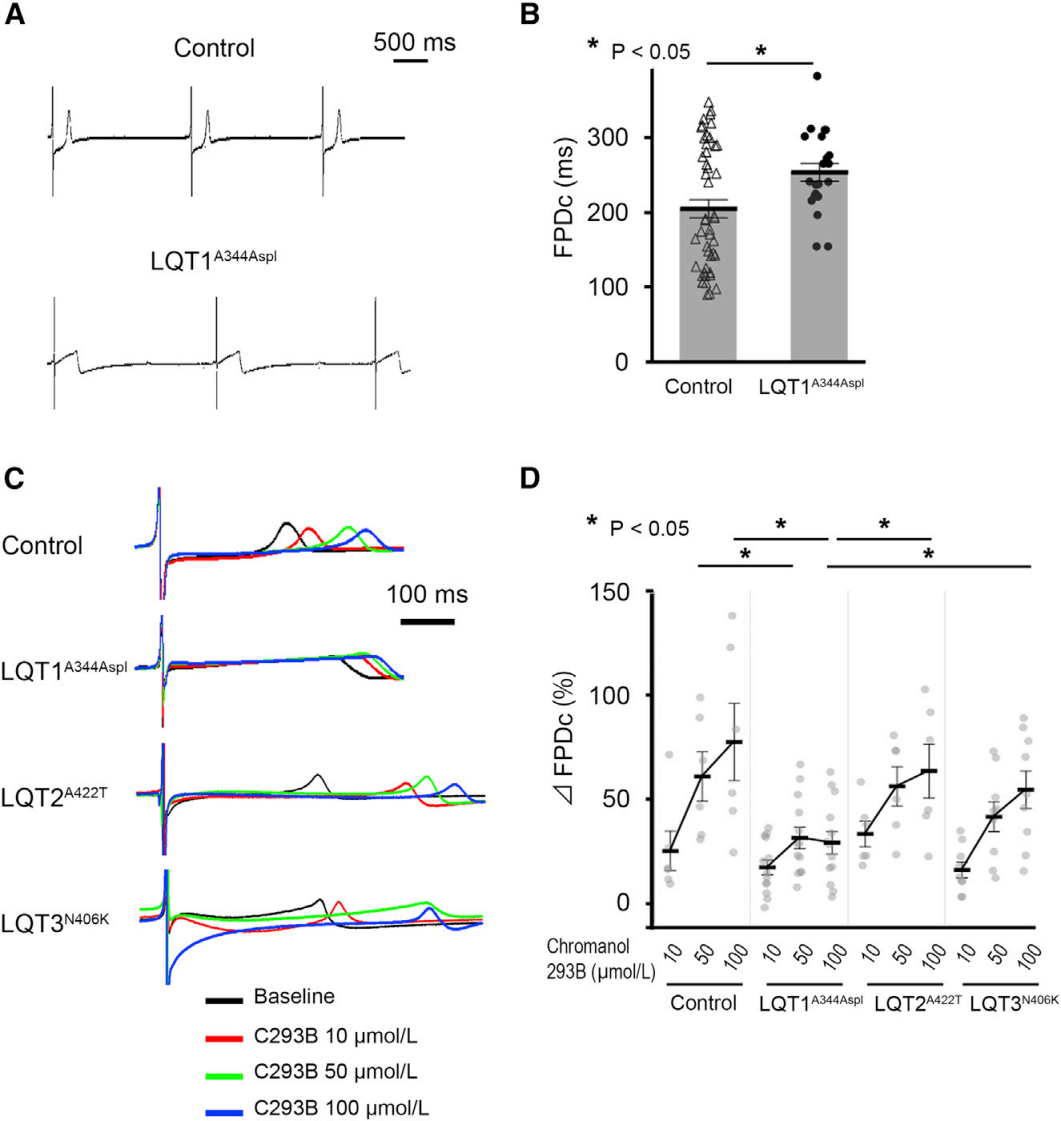
Functional Analysis of iPSC-CMs Using MEA Following I_Ks_ Blockade (A) Representative traces of FP in Control- and LQT1^A344Aspl^-iPSC-CMs. (B) FPDc at baseline was longer in LQT1^A344Aspl^-iPSC-CMs than in Control-iPSC-CMs (independent experiments, n = 45 and 21 from independent differentiation experiments, n = 16 and 6 in Control- and LQT1^A344Aspl^-iPSC-CMs, respectively; mean ± SEM; p < 0.05; unpaired Student's t test). ^∗^p < 0.05. (C) Representative traces of the FPD following administration of 10 μmol/L (red), 50 μmol/L (green), and 100 μmol/L (blue) chromanol 293B in Control- and LQT1^A344Aspl^-iPSC-CMs. (D) Averaged %ΔFPDc in each cell line. %ΔFPDc upon treatment with 100 μmol/L chromanol 293B was significantly smaller in LQT1^A344Aspl^-iPSC-CMs than in Control-, LQT2^A422T^-, and LQT3^N406K^-iPSC-CMs (independent experiments, n = 6, 13, 6, and 9 from independent differentiation experiments, n = 3, 5, 3, and 3 in Control-, LQT1^A344Aspl^-, LQT2^A422T^-, and LQT3^N406K^-iPSC-CMs, respectively; mean ± SEM; p = 0.001; two-way repeated measures ANOVA). ^∗^p < 0.05; Fisher's LSD post hoc test.

We then performed I_Ks_ blockade to elucidate I_Ks_ deficiency by using different concentrations of chromanol 293B. At baseline, the FPDc of LQT1^A344Aspl^-iPSC-CMs was longer than that of Control-iPSC-CMs (249.4 ± 14.0 ms and 202.2 ± 10.8 ms, respectively; p < 0.05) (Figures 4A and 4B). Figure 4C shows representative FP traces in each CM group treated with 10, 50, and 100 μmol/L chromanol 293B. Upon administration of 100 μmol/L chromanol 293B, LQT1^A344Aspl^-iPSC-CMs showed a significantly smaller %ΔFPDc than those observed in Control-, LQT2^A422T^-, and LQT3^N406K^-iPSC-CMs (Figure 4D). These results suggested that the effect of I_Ks_ blockade on FPD prolongation reflected the I_Ks_ density. Regarding the FPDc value, LQT2^A422T^-iPSC-CMs exhibited the longest FPDc among the three lines at 100 μmol/L chromanol 293B (Figure S1B).

### LQT3^N406K^-iPSC-CMs Display a Greater Response to I_Na_ Blockade, which Was Normalized by Gene Correction

**Figure 5:**
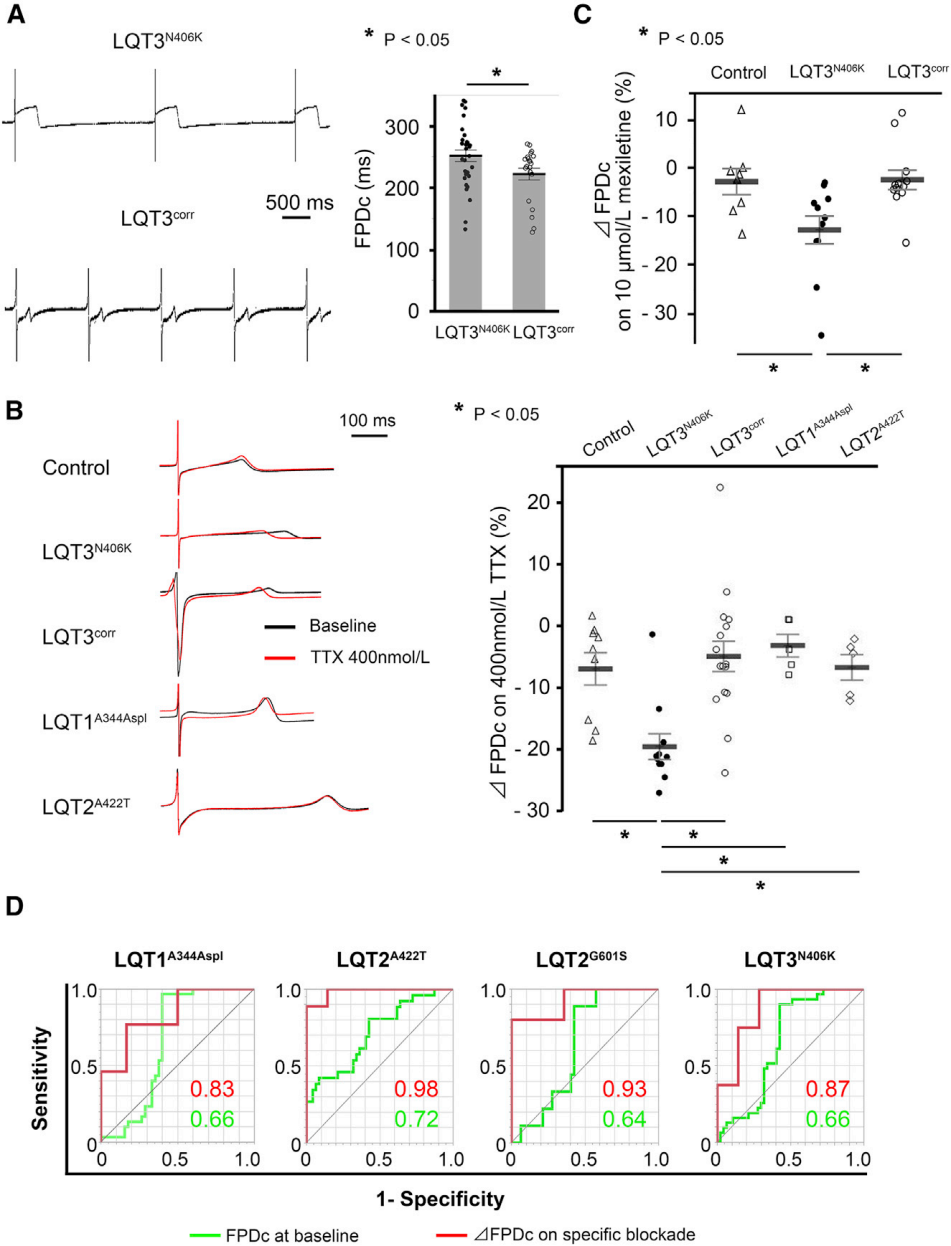
Functional Analysis of iPSC-CMs Using MEA Following I_Na_ Blockade (A) Representative traces of FP in LQT3^N406K^- and LQT3^corr^-iPSC-CMs (left). FPDc at baseline in LQT3^N406K^-iPSC-CMs was significantly shortened by gene correction (right) (independent experiments, n = 36 and 29 from independent differentiation experiments, n = 8 and 8 in LQT3^N406K^- and LQT3^corr^-iPSC-CMs, respectively; mean ± SEM; p < 0.05; unpaired Student's t test). ^∗^p < 0.05. (B) Representative traces of the FP following administration of 400 nmol/L (red) TTX in Control-, LQT3^N406K^-, LQT3^corr^-, LQT1^A344Aspl^-, and LQT2^A422T^-iPSC-CMs (left). The response to treatment with 400 nmol/L TTX was significantly larger in LQT3^N406K^-iPSC-CMs than in Control-, LQT3^corr^-, LQT1^A344Aspl^-, and LQT2^A422T^-iPSC-CMs (right) (independent experiments, n = 9, 11, 17, 5, and 5 from independent differentiation experiments, n = 4, 5, 4, 3, and 3 in Control-, LQT3^N406K^-, LQT3^corr^-, LQT1^A344Aspl^-, and LQT2^A422T^-iPSC-CMs, respectively; mean ± SEM; p < 0.001; one-way ANOVA). ^∗^p < 0.05; Fisher's LSD post hoc test. (C) Averaged %ΔFPDc on 10 μmol/L mexiletine in Control-, LQT3^N406K^-, and LQT3^corr^-iPSC-CMs (independent experiments, n = 7, 11, and 12 from independent differentiation experiments, n = 3, 3, and 5, respectively; mean ± SEM; p < 0.001; one-way ANOVA). ^∗^p < 0.05; Fisher's LSD post hoc test. (D) Comparison of AUCs for baseline FPDc and %ΔFPDc upon specific current blockade for recognizing disease-specific iPSC-CMs. Specific current blockade enhanced the accuracy of recognizing disease-specific iPSC-CMs. See also Table S1.

We then blocked I_Na_ using tetrodotoxin (TTX) in order to elucidate the excessive I_Na-Late_ response. At baseline, the FPDc of LQT3^N406K^-iPSC-CMs was longer than that of the corrected LQT3-iPSC-CMs (LQT3^corr^-iPSC-CMs) (Figure 5A). Figure 5B shows representative FP traces in each CM group treated with 400 nmol/L TTX. Upon administration of 400 nmol/L of TTX, the %ΔFPDc for Control-, LQT3^N406K^-, LQT3^corr^-, LQT1^A344Aspl^-, and LQT2^A422T^-iPSC-CMs was –6.8 ± 2.8%, –19.0 ± 2.6%, –4.8 ± 2.0%, –3.0 ± 3.8%, and –6.6 ± 3.6% (p < 0.05), respectively. A similar response was observed using mexiletine, another typical I_Na_ blocker (Figure 5C).

### %ΔFPDc on Specific Current Blockade Is a Better Predictor for Estimating LQTS Subtype than Baseline FPDc in the iPSC-CM Model

To determine the accuracy of specific current blockade, receiver operating characteristic (ROC) curve analysis was performed in each cell line. Although the area under the ROC curves (AUCs) for baseline FPDc were <0.75, the AUCs for %ΔFPDc following specific current blockade were significantly improved, indicating that specific current blockade enhanced the detectability of the abnormal current (Figure 5D; Table S1).

## Discussion

Among >15 different LQTS subtypes based on responsible pathogenic genes (Schwartz et al., 2013), LQT1, LQT2, and LQT3 are the most common. In LQT1, exercise or emotional stress often triggers arrhythmias, with β blockers the most effective at preventing these arrhythmic events (Moss et al., 2000, Priori et al., 2004, Schwartz et al., 2001). By contrast, in LQT2, drugs or hypopotassemia trigger TdP, and β blockers are less effective than in LQT1. In LQT3, TdP is triggered during sleep, and mexiletine represents a therapeutic option based on its suppression of excessive I_Na-Late_. Therefore, precise classification of LQTS subtypes is clinically important for determining therapeutic strategies and predicting prognosis (Inoue and Yamanaka, 2011). In clinical settings, genetic testing is often performed to assist clinical diagnosis of LQTS; however, it identifies variants in LQTS-related genes in only 60% of cases (Kapa et al., 2009, Wehrens et al., 2002). In addition, not all identified variants are proven to be causative, with many having unknown pathogenicity in the absence of information regarding their electrophysiological consequences (VUSs). Moreover, abnormalities caused by variants in intronic or promoter regions might be overlooked by genetic testing alone. Therefore, a patient-oriented diagnostic system based on its specific phenotype plays an important role in compensating for the shortcomings of genetic testing.

In this study, we successfully distinguished LQT1, LQT2, and LQT3 based on the phenotype displayed by iPSC-CMs to specific current blockade. In addition, we demonstrated that this could be performed using MEA for increased accessibility to the method, as well as its enabling higher throughput, which is optimal for screening systems. To verify the accuracy of this protocol, we generated an ROC curve showing significantly improved %ΔFPDcs upon specific current blockade as compared with FPDcs acquired at baseline, thereby supporting the ability of the method to recognize disease-specific iPSC-CMs. For LQT2, we performed voltage-clamp and current-clamp assays to support the MEA data, and confirmed that gene correction recovered I_Kr_ density, which correlated with the normalized I_Kr_ blockade response of the FPDc in LQT2^corr^-iPSC-CMs. Consistent with these results, Holzem et al. (2016) demonstrated that the reduced I_Kr_ blockade response reflected reduced I_Kr_ expression, as shown via optical imaging of perfused left-ventricular wedge preparations in patients with heart failure. In addition to LQT2, we also clarified that LQT1^A344Aspl^-iPSC-CMs displayed an attenuated response to I_Ks_ blockade, and that LQT3^N406K^-iPSC-CMs displayed a greater response to I_Na_ blockade, which was normalized by gene correction. Of significant note, each type of LQT-iPSC-CMs showed specific response which reflected the impaired ion currents as follows: LQT1^A344Aspl^-iPSC-CMs showed smaller %ΔFPDc upon I_Ks_ blockade while they showed normal %ΔFPDc upon I_Kr_ blockade. In contrast, LQT2^A422T^-iPSC-CMs showed smaller %ΔFPDc upon I_Ks_ blockade while they showed normal %ΔFPDc upon I_Kr_ blockade. These results strongly suggested that our system has a potential to distinguish the subtypes of LQTS. Regarding I_Ks_ blockade, no significant difference in %ΔFPDc on 10 μmol/L chromanol 293B was observed. Given the half maximal inhibitory concentration for KCNQ1/KCNE1 is 16.1 ± 1.8 μmol/L (Bett et al., 2006), I_Ks_ was only partially blocked. We speculated that small portion of I_Ks_ blockade might be masked by repolarization reserve in iPSC-CMs.

In addition, upon I_Kr_ blockade, some of the LQT2^A422T^-iPSC-CMs showed triggered activities at baseline or 30 nmol/L E4031, whereas no arrhythmic events were recorded in other clones, potentially because, compared with the other clones including LQT2^G601S^-iPSC-CMs, LQT2^A422T^-iPSC-CMs had less repolarization reserve composed of ion currents except I_Kr_. Moreover, this might indicate why, upon I_Kr_ blockade, LQT2^G601S^-iPSC-CMs were less vulnerable to EADs than LQT2^A422T^-iPSC-CMs. Interestingly, these results seem to be consistent with the clinical history of donor patients, in which, while the *KCNH2* p.G601S carrier remained asymptomatic, the *KCNH2* p.A422T carrier had episodes of syncope. Further investigation of various mutations, including both pathogenic and non-pathogenic variants, is needed to elucidate this relationship.

Development of iPSC technology has provided increased opportunity for investigating monogenic disorders, such as LQTS, and iPSC-CMs have proven capable of recapitulating clinical phenotypes. However, most previous experiments were performed using labor-intensive techniques, such as patch-clamp methods. On the other hand, MEA, which is also used in neuronal science, improves the throughput of electrophysiological examination and enables CMs to exist in a less-invasive state relative to patch clamping. Therefore, protocols combining iPSC-CMs and the MEA system could potentially offer a less labor-intensive and higher-throughput method for analyzing disease-specific human CMs and could potentially lead to applications enabling clinical diagnosis of LQTS.

This study might offer novel insight into the utility of iPSC-CMs for phenotype-based diagnosis of LQTS. This method has several powerful advantages. First, although next-generation sequencing technology provides information about a large number of rare genetic variants, the pathophysiological significance of such variants is often uncertain. Therefore, the method presented in this study might be helpful in uncovering pathophysiological mechanisms for individuals, especially patients with VUSs or compound mutations, as well as genotype-negative patients. Second, this method enables a more comprehensive and practical diagnosis of LQTS. I_Kr_-mediated LQTS includes LQT2, as well as LQT6 (potassium voltage-gated channel subfamily E regulatory subunit 2), whereas I_Ks_-mediated LQTS includes not only LQT1 but also LQT5 (potassium voltage-gated channel subfamily E regulatory subunit 1) and LQT11 (A-kinase anchoring protein 9), as well as I_Na_-mediated LQTS, including LQT3, 9 (caveolin 3), LQT10 (sodium voltage-gated channel beta subunit 4), and LQT12 (syntrophin alpha 1). Therefore, this current-centric classification^4^ provides us with the clinical ability for selective treatment. For example, mexiletine should be recommended for patients with I_Na_-mediated LQTS, whereas β blockers would be predicted to be effective in all patients with I_Ks_-mediated LQTS. To reveal whether this protocol is more reliable, it is necessary to evaluate the accuracy of this protocol using other cell lines. Nevertheless, the protocol presented here offers important clinical implications in the diagnosis and treatment of LQTS.

This study certainly includes some limitations. We applied Fridericia's formula for FPD correction. However, it is not clear whether the correction formula was valid for iPSC-CMs or not. We used only a small number of iPSC lines with already known pathogenic mutations and did not test iPSCs from LQTS patients carrying VUSs or mutations in the non-coding regions, or patients with no detected mutations. We should also use iPSC lines from persons, without phenotypes, despite carrying pathogenic mutations. In addition, the lack of isogenic controls for LQT1^A344Asppl^ line, the lack of blind tests, the comparisons not performed at the same day of culture, and the variable quality and purity of the iPSC-CMs should be noted as limitations in this study. As such, this study is only a prototype and further investigation is required.

In conclusion, this study showed that the multiple subtypes of LQT could be potentially distinguished by specific ion-channel blockade using the MEA system with patient-derived iPSCs, and that this protocol might serve as a novel method to compensate for the shortcomings of genetic testing of LQTS patients, especially in patients who have VUSs or no identified mutations.

## Experimental Procedures

### iPSC Generation, and CM Differentiation and Purification

iPSCs were generated from LQT patients and healthy controls, as reported previously (Spencer et al., 2014, Wuriyanghai et al., 2018). All protocols were approved by the Committee on Human Research at Kyoto University (Kyoto, Japan) and conformed to the principles of the Declaration of Helsinki. iPSCs were differentiated into CMs using the previously described “GiWi” protocol (Figure S3A) (Lian et al., 2013). Differentiated CMs were purified in glucose-depleted lactate medium, as described previously (Tohyama et al., 2013), and iPSC-CMs were analyzed on days 60 through 100 (Figure S3B). Details are shown in the Supplemental Information.

### Genome Editing

The target site was set to cover the mutation site, and single-guide RNA was constructed. Annealed oligo was inserted into the PX459 (Addgene, Cambridge, MA, USA) plasmid digested by *Bpi*I. The targeting vector was designed to include a floxed puromycin-resistant drug cassette flanked by 1.5-kb homology arms, the 3′ arm of which included the gene-correcting site. The targeting vector was constructed by assembling PCR products of the homology arms, drug-resistant cassette, and backbone pENTR vector with Gibson Assembly (NEB, Ipswich, MA, USA). Thereafter, several point mutations were inserted to avoid further digestion of the targeting site.

The procedures associated with introduction of the vectors were performed, as reported previously (Li et al., 2016). In brief, 5 μg of the CRISPR/Cas9 vector and the targeting vector was introduced into 1.0 × 10^6^ iPSCs using an NEPA 21 electroporator (NEPA GENE, Ichikawa, Japan). Several days after electroporation, drug selection was initiated with 0.7 mg/mL puromycin. Puromycin-resistant cells were dispersed into single cells and dissociated onto a dish. Several days thereafter, single colonies were picked and screened by PCR. Drug-cassette-positive cells were then expanded, and their floxed sites were removed by Cre excision. Sanger sequencing was performed to confirm the sequence of the targeted site and possible off-target sites. Primers used for genome editing are listed in the Supplemental Information (Tables S2 and S3).

### Electrophysiological Recordings

#### MEA Recording

CMs were dissociated with 1 mg/mL collagenase B (Roche, Roswell, GA, USA) and Accumax (Innova Cell Technologies, San Diego, CA, USA). CM suspension (2 μL of 1.5 × 10^4^ cells/μL) was placed onto the Matrigel-coated electrode (MED-P515A; Alpha MED Scientific, Osaka, Japan) (Asakura et al., 2015). After several hours, 1 mL RPMI/B27 medium containing 10% fetal calf serum was added. After 2 to 3 days, the medium was replaced with RPMI/B27 medium. After 6 to 20 days, the FP of spontaneously beating CMs was recorded. FP signals were digitally sampled at 20 kHz through 0.1-Hz high-pass and 10-kHz low-pass filters using the MED64 system (Alpha MED Scientific). FPD was defined as the interval between a positive or negative spike and a subsequent positive deflection, and inter-spike interval (ISI) was defined as the interval between adjacent spikes. These parameters were automatically measured and analyzed using Möbius QT (Alpha MED Scientific). More than 30 beats were recorded, and the FPDs and ISIs of the final 30 beats were averaged, as described previously (Asakura et al., 2015), which was standardized by Fridericia's formula (FPDc = FPD/ISI^1/3^) to minimize influence of a wide range of ISIs on FPDs (Table S4). EAD was defined as relatively slow negative spikes during the repolarizing phase, with samples displaying irregular beating excluded when calculating FPD and ISI. EAD-positive samples were defined as showing more than 5 EADs among 30 beats. The prolongation rate of FPDc (%ΔFPDc) was calculated as follows: %ΔFPDc (%) = [(FPDc after blockade – FPDc at baseline)/FPDc at baseline × 100]. For specific current blockade, we used E4031 (Wako Pure Chemicals, Osaka, Japan), chromanol 293B (Sigma-Aldrich, St. Louis, MO, USA), TTX (Alomone Labs, Jerusalem, Israel), and mexiletine (Sigma-Aldrich, Tokyo, Japan). Drug was gently administered by taking half of the medium out, diluting it, and then returning it to the solution. For measurement under stable conditions, FPD was measured 30 to 60 min after drug administration. All the data were acquired from at least three independent experiments.

#### Patch-Clamp Recording

CMs dissociated with Accutase (Thermo Fisher Scientific) were adhered onto Matrigel-coated glass coverslips for 5 days before recording AP in current-clamp mode using the perforated patch technique, whereas I_Kr_ was recorded from single cells in voltage-clamp mode using the ruptured whole-cell patch technique (Ma et al., 2011). The pipette solution comprised (in mM): 150 KCl, 5 NaCl, 2 CaCl_2_, 5 EGTA, 10 HEPES, and 5 MgATP (pH 7.2, adjusted with KOH), with amphotericin B added during AP recording (0.3 mg/mL, final concentration). The extracellular solution comprised (in mM): 150 NaCl, 5.4 KCl, 1.8 CaCl_2_, 1 MgCl_2_, 15 glucose, 15 HEPES, and 1 Na-pyruvate (pH 7.4, adjusted with NaOH), with 2 μM nifedipine added during I_Kr_ recording.

Current-clamp recordings were sampled and filtered at 10 kHz. To evaluate AP prolongation by E4031, I_K1_ current was artificially injected using the dynamic clamp technique, as reported previously (Bett et al., 2013). Detailed dynamic clamp procedures are described in the following section. Ventricular-type CMs were defined as those exhibiting an APD_90_/APD_50_ < 1.4. E4031 administration (100 nM) was performed in ventricular-type CMs.

Before adding nifedipine for I_K_ measurement, AP was recorded to identify ventricular-type CMs. Voltage-clamp recordings were then sampled at 2 kHz and filtered at 1 kHz. The voltage-clamp trace was obtained as follows: after a −40-mV holding pulse, step pulses were applied from −40 to 10 mV in 5-mV increments for 4 s and with a 10-s cycle length. The tail current was measured at the peak immediately after the depolarizing pulse. The measured current was standardized based on the patched cell-membrane capacitance. Therefore, I_Kr_ was defined as the E4031-sensitive current upon addition of 500 nM E4031 to the extracellular solution. Pipettes pulled from thin-walled borosilicate glass capillaries (TW150-4; World Precision Instruments, FL, USA) with a PP-830 puller (Narishige, Tokyo, Japan) exhibited between 4.0 and 7.0 MΩ resistance in voltage clamp and between 3.0 and 5.0 MΩ resistance in current clamp. All recordings were performed at between 35°C and 37°C. Data were acquired with an Axon 700B MultiClamp, Digidata 1440A digitizer hardware, and pCLAMP 10.4 software (Molecular Devices, Sunnyvale, CA, USA), as required. All the data were acquired from at least three independent experiments.

#### Real-Time I_K1_ Injection Using the Dynamic Clamp Technique

As described previously (Bett et al., 2013), an analog and digital I/O board PCIe-DAS1602/16 (Measurement Computing Corporation, Norton, MA, USA) was used to transfer the signal of membrane voltage to the I_K1_ signal using the same source code. In addition, 1 pA/pF of peak I_K1_ was injected to fix the MDP at approximately –80 mV in our iPSC-CMs before and after E4031 administration. I_K1_ was adjusted using a potentiometer (custom-made by Inter Medical, Nagoya, Japan). The I_K1_ equation was optimized for ventricular cells according to previous reports (Bett et al., 2013, Koumi et al., 1995, Van Putten et al., 2015). The equation we used was as follows: I_K1_ = 0.5 × (*Vm* + 85)/(1 + *e*^[0.0896(*Vm*^ ^+ 85)]^).

### Statistical Analysis

JMP Pro 13 (SAS, Cary, NC, USA) was used for statistical analysis. Data are presented as the mean ± SEM. An unpaired Student's t test or one-way analysis of variance (ANOVA), followed by Fisher's least significant difference (LSD) test, was used for two- or more than two-group comparisons. For repetitive measurements, we performed a two-way repeated measures ANOVA, followed by Fisher's LSD test. Pearson's chi-square test was used to determine independence of categorical data. Results were considered statistically significant at p < 0.05.

## Author Contributions

Conceptualization, D.Y., S.B., and T.M.; Methodology, D.Y., S.B., T.M., and T.H.; Investigation, D.Y., H.S., T.H., K.A., K.M., H.K., Y.W., and Y.Y.; Writing –– Original Draft, D.Y.; Writing – Review & Editing, S.B., T.M., K.U., B.R.C., and M.H.; Funding Acquisition, S.B., T.M., and M.H.; Resources, S.B., T.M., K.H., B.R.C., M.H., and T.H.; Supervision, S.B., T.M., M.H., J.T., and T.H.

## Supplemental Information

Document S1. Supplemental Experimental Procedures, Figures S1–S3, Tables S1 and S4

Table S2. Sequences of Primers Used for Genome Editing of LQT2^A422T^-iPSCs, Related to Genome Editing Section

Table S3. Sequences of Primers Used for Genome Editing of LQT3^N406K^-iPSCs, Related to Genome Editing Section

Document S2. Article plus Supplemental Information
