# Supplementary material for: Phenotype-Based High-Throughput Classification of Long QT Syndrome Subtypes Using Human Induced Pluripotent Stem Cells
Source: Stem Cell Reports. 2019 Aug 1;13(2):394–404. doi: 10.1016/j.stemcr.2019.06.007 (PMC6700479; doi:10.1016/j.stemcr.2019.06.007)
Supplement: Document S1. Supplemental Experimental Procedures, Figures S1–S3, Tables S1 and S4 [file mmc1.pdf]

Stem Cell Reports, Volume *13*

Supplemental Information

Phenotype-Based High-Throughput Classification of Long QT Syndrome Subtypes Using Human Induced Pluripotent Stem Cells

Daisuke Yoshinaga, Shiro Baba, Takeru Makiyama, Hirofumi Shibata, Takuya Hirata, Kentaro Akagi, Koichi Matsuda, Hirohiko Kohjitani, Yimin Wuriyanghai, Katsutsugu Umeda, Yuta Yamamoto, Bruce R. Conklin, Minoru Horie, Junko Takita, and Toshio Heike

Supplemental Information

Figure S1

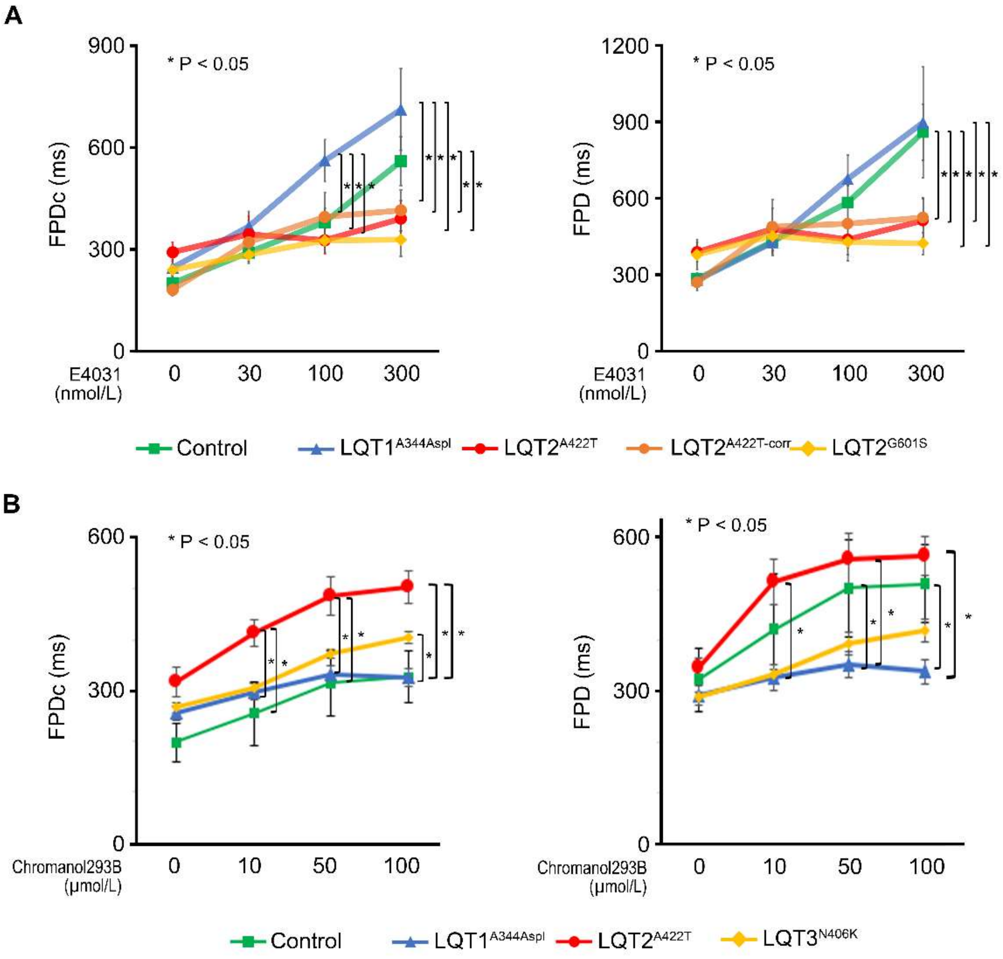

Figure S1. The prolongation of FPDc and FPD upon specific channel-current blockade, related to Figures 2 and 4. (A) Averaged FPDc (left) and FPD (right) before and after E4031 treatment in Control-, $LQT2^{A422T}$-, $LQT2^{A422T\text{-corr}}$-, $LQT2^{G601S}$-, and $LQT1^{A344Aspl}$-iPSC-CMs. FPDc of $LQT2^{A422T}$- and $LQT2^{G601S}$-iPSC-CMs was significantly smaller than that of Control-iPSC-CMs in the presence of 300 nmol/L E4031 and that of $LQT1^{A344Aspl}$-iPSC-CMs in the presence of 100 nmol/L and 300 nmol/L E4031 (independent experiments, n = 23, 24, 12, 9, and 12 from independent differentiation experiments, n = 8, 8, 6, 3, and 3 in Control-, $LQT2^{A422T}$-, $LQT2^{A422T\text{-corr}}$-, $LQT2^{G601S}$-, and $LQT1^{A344Aspl}$-iPSC-CMs, respectively; $P < 0.001$; two-way repeated-measures ANOVA). FPD of $LQT2^{A422T}$- and $LQT2^{G601S}$-iPSC-CMs was significantly smaller than that of Control- and $LQT1^{A344Aspl}$-iPSC-CMs in the presence of 300 nmol/L E4031 (independent experiments, n = 23, 24, 12, 9, and 12 from independent differentiation experiments, n = 8, 8, 6, 3, and 3 in Control-, $LQT2^{A422T}$-, $LQT2^{A422T\text{-corr}}$-, $LQT2^{G601S}$-, and

LQT1^{A344Aspl}-iPSC-CMs, respectively; $P < 0.001$; two-way repeated-measures ANOVA). *$P < 0.05$, Fisher′s LSD post hoc test. Data are represented as mean ± SEM. (B) Averaged FPDc (left) and FPD (right) before and after chromanol293B treatment in Control-, LQT1^{A344Aspl}-, LQT2^{A422T}-, and LQT3^{N406K}-iPSC-CMs. FPD of LQT1^{A344Aspl}-iPSC-CMs was significantly smaller than that of Control-iPSC-CMs in the presence of 100 nmol/L Chromanol293B (independent experiments, n = 6, 13, 6, and 9 from independent differentiation experiments, n = 3, 5, 3, and 3 in Control-, LQT1^{A344Aspl}-, LQT2^{A422T}-, and LQT3^{N406K}-iPSC-CMs, respectively; $P < 0.001$; two-way repeated-measures ANOVA). *$P < 0.05$, Fisher′s LSD post hoc test. Data are represented as mean ± SEM.

Figure S2

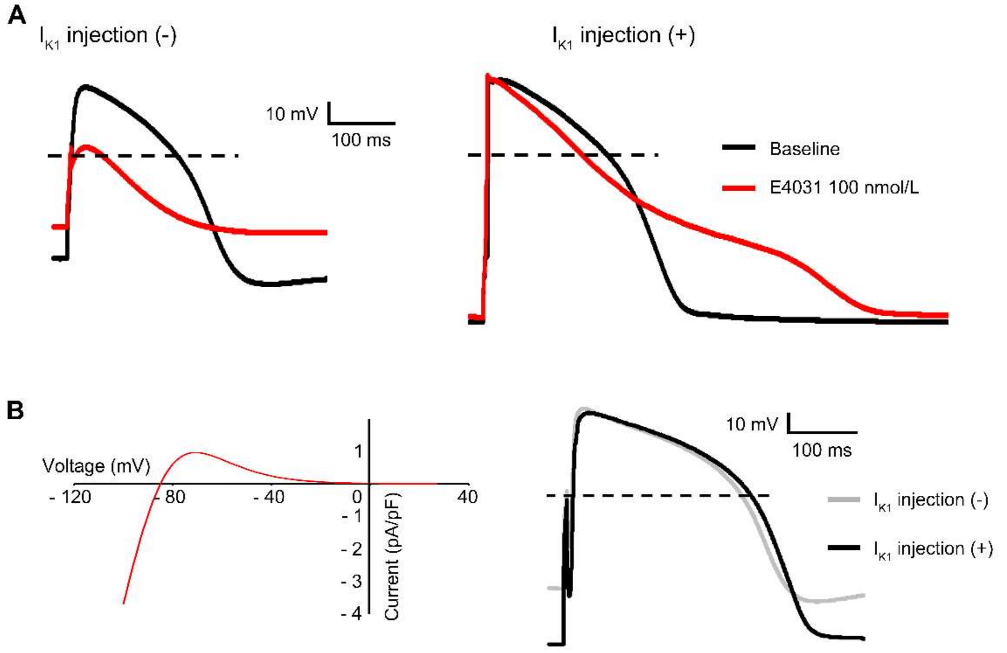

Figure S2. I_{K1} injected through dynamic clamp and AP traces, related to Figure 3. (A) Representative traces of APs at 1-Hz pacing with MDP elevated by I_{Kr} blockade. AP could not be properly assessed due to insufficient depolarization (left). MDP fixation by injection of I_{K1} allowed AP assessment (right). (B) Current-voltage relationship of injected I_{K1} to iPSC-CMs through dynamic clamp (left). AP traces with and without I_{K1} injection (right).

Figure S3

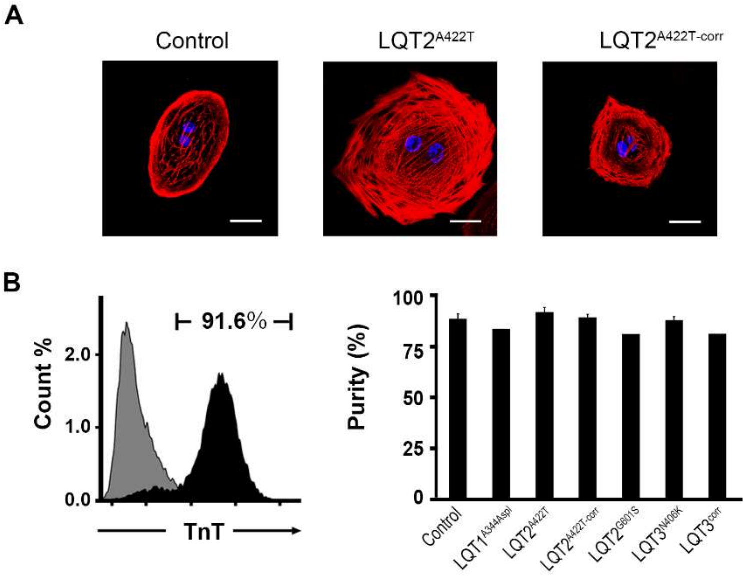

Figure S3. Characterization of iPSC-CMs, related to the Experimental Procedures. (A) Immunofluorescence analysis of cTnT in Control-iPSC-CMs, LQT2^{A422T}-iPSC-CMs, and LQT2^{corr}-iPSC-CMs. Scale bars: 25 µm. (B) Purity of differentiated iPSC-CMs from each cell line. Flow cytometric analysis of CMs stained for cTnT (left, black) or isotype control (left, gray). Averaged percentage of cTnT-positivity was >80% in each cell line (right). Data are represented as mean ± SEM.

cTnT: cardiac troponin T.

Table S1. Optimal cut-off values of ΔFPDcs as determined by sensitivity and specificity at different cut-off values, related to Figure 5.

	Specific current blocker	Concentration	Optimal cut-off value of ΔFPDc (%)	Sensitivity (%)	Specificity (%)
**LQT1^{A344Aspl}**	Chromanol 293B	100 µmo/L	40.9	76.9	83.3
**LQT2^{A422T}**	E4031	300 nmol/L	85.2	77.8	100
**LQT2^{G601S}**	E4031	300 nmol/L	92.9	80.0	100
**LQT3^{N406K}**	tetrodotoxin	400 nmol/L	−2.1	100	71.4

.

Table S2. Sequences of primers used for genome editing of LQT2^{A422T}-iPSCs, related to Genome editing section.

(Please see Excel file)

Table S3. Sequences of primers used for genome editing of LQT3^{N406K}-iPSCs, related to Genome editing section.

(Please see Excel file)

Table S4. Inter-spike intervals in each cell line, related to *MEA recording* section.

Cell line	Number of samples	Inter-spike interval (ms)		P-value (vs. control)
		Average	S.E.	
**Control**	45	3184.2	320.9	-
**LQT1^{A344spl}**	30	1292.5	292.6	< 0.01
**LQT2^{A422T}**	35	2543.3	288.2	0.09
**LQT2$^{A422T\text{-}corr}$**	12	4312.8	991.1	0.04
**LQT2^{G603S}**	9	4301.9	961.4	0.06
**LQT3^{N406K}**	29	1490.9	90.1	< 0.01
**LQT3^{corr}**	36	1804.1	133.5	< 0.01

Supplemental Experimental Procedures

Generation and Culture of iPSCs

Dermal fibroblasts obtained from an LQT2 patient, an LQT3 patient with a history of recurrent syncope, and a healthy control were transduced with retroviruses harboring the transcription-factor genes octamer-binding transcription factor 3/4 (*OCT3/4*), sex determining region Y-box 2 (*SOX2*), Krüppel-like factor 4 (*KLF4*), and *c-MYC* (Spencer et al., 2014). Additionally, from an LQT1 patient, and another LQT2 patient, peripheral blood mononuclear cells were obtained and reprogrammed to iPSCs, transfected with the episomal vectors encoding *OCT3/4*-, *SOX2*-, *KLF4*-, *L-MYC*-, *LIN28*-, and a *TP53*-targeting small-hairpin RNA (Wuriyanghai et al., 2018). All procedures were performed after consent forms were signed by all donors. All protocols were approved by the Committee on Human Research at the University of California, San Francisco (San Francisco, CA, USA) and at Kyoto University (Kyoto, Japan) and conformed to the principles of the Declaration of Helsinki. Characterized iPSCs were maintained in mTeSR1 medium (Stem Cell Technologies, Vancouver, BC, Canada) on Matrigel (BD Biosciences, San Jose, CA, USA)-coated plates at 37°C and 5% CO_2. Two clones from LQTS patients, one clone from the healthy control, and another control clone purchased from RIKEN (201B7; Tokyo, Japan) were used for further experiments.

CM Differentiation and Purification

iPSCs were differentiated into CMs using the "GiWi" protocol (Lian et al., 2013). Briefly, 3 days after iPSCs were seeded onto a 12-well plate, the medium was replaced with RPMI/B27 medium lacking insulin (Thermo Fisher Scientific, Waltham, MA, USA) and containing 12 μM CHIR99021 (Selleckchem, Houston, TX, USA) (Day 0). Exactly 24 h later (Day 1), the medium was replaced with RPMI/B27 medium lacking insulin. On Day 3, the medium was replaced with RPMI/B27 medium lacking insulin and containing 5 μM IWP2 (Tocris Bioscience, Bristol, UK). On Day 5, the medium was replaced with RPMI/B27 medium lacking insulin. On Day 7 and every 3 to 4 days thereafter, RPMI/B27 medium was replenished. CMs were purified in glucose-depleted lactate medium on Days 23 through 29 after differentiation, as previously described (Tohyama et al., 2013). Differentiated CMs were analyzed on Days 60 through 100.

Immunocytochemistry

iPSCs and iPSC-CMs were fixed in 4% paraformaldehyde (Wako Pure Chemical, Osaka, Japan) and permeabilized in 0.1% Tween. Nonspecific binding was blocked by incubation overnight in phosphate-buffered saline containing 3% skim milk. Anti-OCT3/4 (1:50; Santa Cruz Biotechnology, Dallas, TX, USA, ref. no. sc-5279), anti-TRA-1-81 (1:100; Millipore, Billerica, MA, USA, ref. no. MAB4381), and anti-cardiac troponin T (cTnT) (1:100; Thermo Fisher

Scientific, ref. no. MA5-12960) primary antibodies were used. The secondary antibodies were Alexa Fluor 568-conjugated donkey anti-mouse IgG (1:1000; Life Technologies, Waltham, MA, USA, ref. no. A10037), Alexa Fluor 488-conjugated donkey anti-mouse IgG (1:1000; Life Technologies, ref. no. A21202), and Cy3-conjugated donkey anti-goat IgM (1:100; Jackson ImmunoResearch, West Grove, PA, USA, ref. no. 715-166-020). BZ9000 (Keyence, Osaka, Japan) and TCS-SP8 (Leica, Nussloch, Germany) microscopes were used to image iPSCs and iPSC-CMs, respectively.

Flow cytometry

iPSC-CMs were fixed in BD Cytofix/Cytoperm (BD Biosciences, San Jose, CA, USA) for 20 min, washed with BD Perm/Wash, stained with an anti-cTnT antibody (1:100; Thermo Fisher Scientific), and labeled with Alexa Fluor 488-conjugated donkey anti-mouse IgG (1:1000; Life Technologies, ref. no. A21202). Data were acquired using a FACS Verse flow cytometer and analyzed using FACSuite software (BD Biosciences).
